# An algorithm for predicting job vacancies using online job postings in Australia

**DOI:** 10.1057/s41599-023-01562-9

**Published:** 2023-03-13

**Authors:** David Evans, Claire Mason, Haohui Chen, Andrew Reeson

**Affiliations:** CSIRO, Canberra, ACT Australia

**Keywords:** Economics, Economics

## Abstract

Timely and accurate statistics on the labour market enable policymakers to rapidly respond to changing economic conditions. Estimates of job vacancies by national statistical agencies are highly accurate but reported infrequently and with time lags. In contrast, online job postings provide a high-frequency indicator of vacancies with less accuracy. In this study we develop a robust signal averaging algorithm to measure job vacancies using online job postings data. We apply the algorithm using data on Australian job postings and show that it accurately predicts changes in job vacancies over a 4.5-year period. We also show that the algorithm is significantly more accurate than using raw counts of job postings to predict vacancies. The algorithm therefore offers a promising approach to the timely and reliable measurement of changes in vacancies.

## Introduction

Job vacancies are an important measure of the tightness of labour markets (Blanchard et al., 1989; Elsby et al., 2015; U.S Bureau of Labor Statistics, 2022). In the short run, changes in the number of job vacancies reflect changes in firms’ demand for workers, with higher (lower) demand leading to increases (decreases) in the number of vacancies (Krumel et al., 2021; U.S Bureau of Labor Statistics, 2022). In the long run, changes in the number of job vacancies can also reflect changes in labour supply, with increases (decreases) in labour supply leading to decreases (increases) in the number of unfilled vacancies (Duval et al., 2022; Holt & Martin, 1966; Krumel et al., 2021).

Central banks and governments monitor job vacancies and other employment data to understand labour market conditions and inform decisions regarding monetary and fiscal policy (DiCecio & Gascon, 2009; Edwards & Gustafsson, 2013). In times of crisis, such as the labour market shock in 2020 associated with the COVID-19 pandemic, governments use job vacancies data (along with other indicators of labour demand) to determine the scale, duration, and timing of income replacement programmes for workers (Autor et al., 2022; Bishop & Day, 2020; Cotofan et al., 2021). In these scenarios, timely and reliable data on the labour market enable policymakers to understand current conditions and adapt their policies accordingly.

The traditional approach to measuring job vacancies involves surveying employers. Many national statistical agencies conduct periodic surveys in which they ask employers about their number of job vacancies. For example, the US Bureau of Labor Statistics conducts the Job Openings and Labor Turnover Survey (U.S Bureau of Labor Statistics, 2022), the UK Office for National Statistics conducts the Vacancy Survey (Office for National Statistics, 2012), and the Australian Bureau of Statistics runs the Job Vacancies Survey (ABS, 2022). In each of these surveys, the statistical agency asks a representative sample of employers to report their number of vacancies, and then uses these reported values to estimate the total number of vacancies. These estimates are highly accurate but are reported infrequently, at a high level of temporal aggregation, and with time lags. For example, the US Bureau of Labor Statistics reports monthly estimates of vacancies with a one-month lag (U.S Bureau of Labor Statistics, 2022), the UK Office for National Statistics reports rolling quarterly estimates of vacancies with up to a six-week lag (Office for National Statistics, 2012), and the Australian Bureau of Statistics reports quarterly estimates of vacancies with a 6-week lag (ABS, 2022). These high levels of temporal aggregation (monthly, quarterly) and time lags in reporting can delay the detection of changes in labour market conditions, which can in turn delay policy responses to the new conditions. Such delays are problematic during periods of economic volatility where governments seek to adapt their policies to rapid changes in labour demand.

Online job postings provide an alternative measure of job vacancies. Since online job postings are high frequency, they can provide an earlier signal of changes in vacancies than official vacancies statistics (Office for National Statistics, 2021; Krumel et al., 2021). The drawback is that online job postings provide an unrepresentative sample of the population of job vacancies. This unrepresentativeness arises for several reasons. First, not all job vacancies are advertised (Carnevale et al., 2014; Kureková et al., 2015; Office for National Statistics, 2021). Second, each online platform that hosts job postings provides incomplete coverage of the population of job postings, as not all employers post their vacancies on the platform (Office for National Statistics, 2021). Platforms scrape postings from each other to improve their coverage, but these scraping processes likely miss certain platforms (e.g., small, emerging ones) and are unable to scrape others due to technical and legal protections, so coverage remains incomplete (Zhao et al., 2021). Third, since recruiters often post job postings to multiple platforms and platforms scrape postings from each other to improve coverage, duplicate postings are common (Jijkoun, 2016; Office for National Statistics, 2021; Zhao et al., 2021). Fourth, one job posting can be used to fill multiple positions (i.e., represents multiple vacancies) (Carnevale et al., 2014; Office for National Statistics, 2021). Fifth, platforms’ imperfect data cleaning processes mean that job postings can remain in their databases after the vacancy has been filled. Finally, online job postings over-represent specific types of workers (Beresewicz et al., 2019; Hershbein & Kahn, 2018), making them overly responsive to some localised labour market changes and insufficiently responsive to others. Each of these factors causes the count of job postings to differ from the actual number of job vacancies. As such, we expect the two measures to be positively correlated over time, rather than perfectly aligned.

**Fig. 1:**
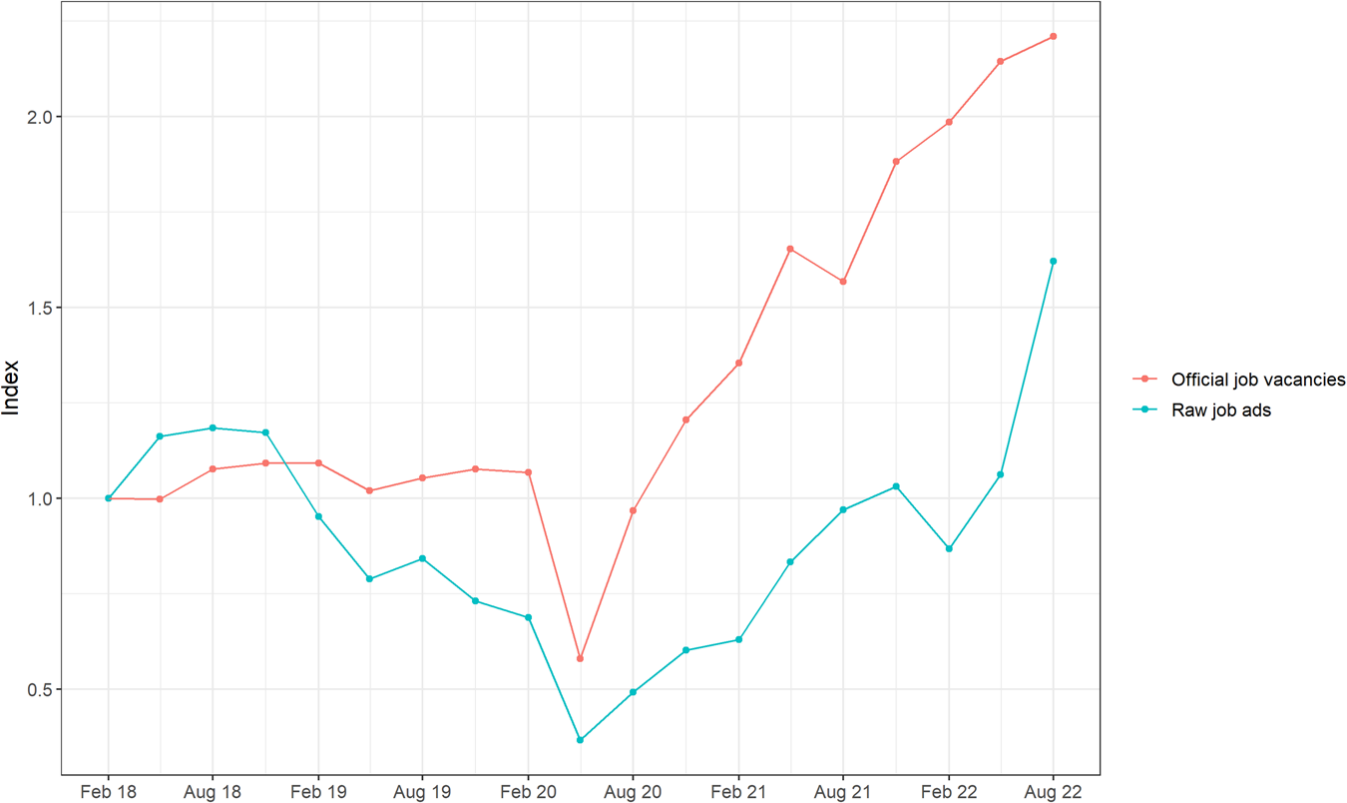
Australian job vacancies and job postings over time. Indexes of quarterly Australian job vacancies (from the Job Vacancies Survey) and Adzuna Australia job postings between February 2018 and August 2022.

Figure 1 illustrates the differences between the number of vacancies and the number of job postings for Australia between 2018 and 2022. The number of vacancies is drawn from the Job Vacancies Survey, which provides estimates of the total stock of vacancies on each survey date. The number of new job postings is sourced from Adzuna Australia, an online search engine that collates job postings from hundreds of sources, such as employers’ websites and online job boards (Zhao et al., 2021). To align the number of new postings (a flow variable) with the number of vacancies (a stock variable), we use the total number of new postings in the six weeks preceding the survey date as a proxy indicator of the number of vacancies on the survey date. In Fig. 1 the vacancies and postings series have both been indexed to a value of 1 in the February 2018 quarter to illustrate how they differ over time (see the Supplementary Information for raw counts). The figure shows that there is only moderate alignment between changes in job postings and changes in vacancies over this period (these two variables have a correlation coefficient of 0.65). The two measures are well aligned throughout 2018, but in 2019 the job postings data indicate a decline in vacancies when no such decline was reported in the official job vacancy statistics. The job postings data also indicate only a gradual increase in vacancies after May 2020 when the official statistics show a larger increase in vacancies. There are several potential drivers of this misalignment between postings and vacancies over time, including Adzuna (and the platforms it scrapes from) improving their deduplication procedures, larger platforms preventing other platforms from scraping their postings, and reduced recruiter activity (leading to fewer duplicate postings).

The objective of this study is to develop an accurate and timely indicator of job vacancies using job postings data. We use Adzuna Australia job postings data to develop and evaluate this indicator. Instead of using the total count of new job postings from all the sources that Adzuna Australia collates to measure vacancies, our method treats each source’s percentage change in new job postings from one period to the next as an equally weighted signal of the percentage change in vacancies. The method then averages this ensemble of signals to estimate the change in vacancies. The rationale behind this approach is to reduce the influence of sources with large counts of postings on estimates, as these sources are more likely to scrape postings from many other sources and therefore more likely to suffer from duplicates and other inaccuracies (Office for National Statistics, 2021). To evaluate our method, we compare its predictions to official estimates of job vacancies over a 4.5-year period.

## Related work

Various organisations and researchers have explored using online job postings as a high-frequency measure of temporal changes in labour demand, particularly following the onset of the COVID-19 pandemic in 2020 (OECD, 2021; Office for National Statistics, 2021). Forsythe et al. (2020) used Burning Glass data on new job postings to show that labour demand contracted sharply between February and April 2020 with the onset of the pandemic. Krumel et al. (2021) demonstrated that Forsythe et al. (2020) overstated the contraction in labour demand by focusing on changes in new job postings and that changes in the total stock of postings indicated a smaller (but still significant) contraction in labour demand. This study highlighted the importance of accounting for changes in the stock of unfilled postings, not just the flow of new postings, in measuring total labour demand over time, and showed that increases in the time required to fill vacancies (rather than increases in new postings) were the main driver of the increase in total postings in the US between mid-2020 and May 2021.

Several studies have reviewed the utility of online job postings in measuring aspects of the labour market (Carnevale et al., 2014; Duenser & Mason, 2020; Kurekova et al., 2015; Office for National Statistics, 2021). These studies highlight that the main benefit of job postings data relative to traditional survey data is that (as big data) they enable the analysis of labour demand at detailed regional, occupational, skillset, and sector levels. The studies also highlight the shortcomings of online job postings as a measure of labour demand, such as their lack of representativeness, as discussed in the Introduction.

To address the lack of representativeness of online job postings data, researchers have developed methods to calibrate job postings to official statistics (Beresewicz et al., 2019; Cammeraat & Squicciarini, 2021; Turrell et al., 2019). For example, Turrell et al. (2019) reweight their database of job postings to make the proportion of postings in each sector align with the sectoral composition of vacancies according to the official (Office for National Statistics) Vacancy Survey. The authors then use the calibrated job postings to produce estimates of labour market tightness at detailed spatial and occupational levels. Similarly, Beresewicz et al. (2019) calibrate job postings data to official vacancy data collected by Statistics Poland to produce representative estimates of skills demand. These studies use already-published official data on job vacancies to improve the representativeness of online job postings data at given points in time. In contrast, our study is focused on developing a method that uses job postings data to accurately predict the official vacancy counts ahead of time (and provide a high-frequency measure of vacancies).

## Methods

### Data

This research is based on data provided by Adzuna Australia. This database contains job postings that employers and recruitment agencies post directly on Adzuna Australia’s online platform, along with job postings that Adzuna Australia scrapes from thousands of other sources (e.g., larger employers’ websites) (Zhao et al., 2021). The database contains 8,460,963 new job postings that were posted within our study period (2017 to 2022). Adzuna Australia’s scraping process increases the database’s coverage of the population of job postings but also increases the likelihood of duplicate postings entering the database (Zhao et al., 2021). To address this problem, Adzuna Australia screens the postings it scrapes from other sources and removes suspected duplicates (Duenser & Mason, 2020). We then apply an additional filter to remove other suspected duplicates (postings with the same job title, same location, similar posting dates, and near-identical job descriptions) (Zhao et al., 2021). Once we have filtered out these suspected duplicates, we use the remaining postings to compute the weekly counts of new postings. Adzuna Australia records the source of each job posting (the website or recruitment agency that the job posting was captured from) as a field in their database.

We source official job vacancy counts from the Australian Bureau of Statistics Job Vacancies Survey (ABS, 2022). This survey asks a representative sample of Australian employers to report their number of vacancies as at the survey date. These reported vacancies are then weighted up to estimate the total number of vacancies at that point in time. The survey is conducted on the middle Friday of February, May, August, and November. Estimates are then published at the end of the quarter (e.g., the estimate of the number of vacancies in the middle of February is published on 31 March). The survey has high response rates (over 95%) and provides accurate estimates of the true number of vacancies (e.g., in the November 2021 quarter the estimated vacancy count was 396,100, with a standard error of 11,200) (ABS, 2022). The average number of job vacancies across the study period was 271,000, with a minimum of 124,500 in May 2020 and a maximum of 475,000 in August 2022.

Job vacancies and job postings, as defined above, are different statistical concepts. The count of job vacancies is measured at a point in time, making it a stock variable. The count of new job postings is measured per unit of time (new postings per week), making it a flow variable. In our analysis we use the total number of new job postings in the six weeks preceding date of the Job Vacancies Survey as a proxy indicator of the number of vacancies on that date. These measures are related in that an increase in the flow of new postings in the weeks preceding time *t* increases the stock of vacancies at time *t*. The measures are also related in that increases in the time required to fill vacancies (e.g., due to tightening of the labour market) increases both the stock of vacancies and the flow of new postings into Adzuna’s database (without affecting the true flow of new postings). Increases in the time required to fill vacancies increases the flow of new postings to Adzuna’s database because Adzuna deletes postings from its database after 60 days; if these postings are unfilled and continue to be posted on websites, Adzuna then scrapes them back into its database and counts them as new postings. Further, increases in the time required to fill vacancies will lead to some employers rewording their unfilled postings, with Adzuna then scraping the reworded postings into its database as new postings. In the Supplementary Information we use simulation to verify that the flow of new postings to Adzuna’s database is positively correlated with the stock of vacancies when the time required to fill vacancies changes.

### An ensemble approach to measuring changes in job vacancies

The total job postings captured by Adzuna Australia implicitly weights each source’s (website’s or recruiter’s) signal according to the change in the source’s count of postings, making it highly responsive to changes in sources with large counts of postings and relatively unresponsive to changes in sources with small counts of postings. This aspect of the measure can be problematic in that sources with large counts of postings are likely to suffer from duplicate postings, as recruiters often submit a job posting to several of the most popular platforms and these platforms often scrape postings from each other (Office for National Statistics, 2021; Zhao et al., 2021). When one jobs board wins the tender to post job vacancies for a large employer and they happen to be one of the organisations that do not allow job ads to be scraped from their platform it can create additional noise in the data. As such, changes in the count of postings at the larger platforms might reflect changes in recruiters’ posting behaviour and the platforms’ scraping procedures rather than underlying changes in vacancies (Office for National Statistics, 2021). Meanwhile, sources with relatively small counts of postings might provide reasonably accurate signals of changes in vacancies but have minimal influence on the measure.

**Fig. 2:**
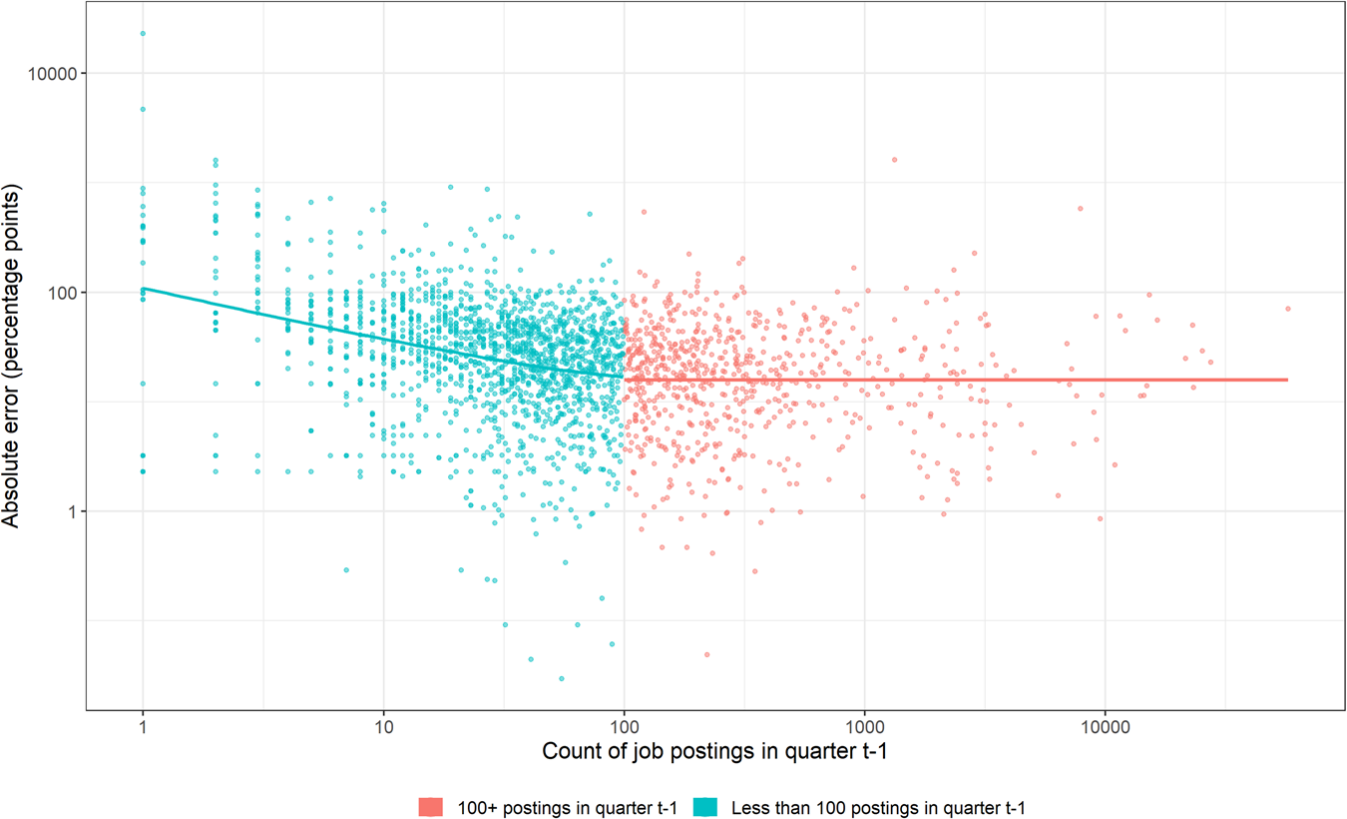
The accuracy of different job postings sources. Absolute prediction errors of sources with different counts of job postings.

To test this hypothesis, we compare the accuracy of all the sources that Adzuna Australia collates in predicting changes in job vacancies across four quarters (May 2017 to February 2018). Figure 2 shows each source’s number of job postings and absolute prediction error (the absolute value of the difference between the source’s percentage change in postings and the actual percentage change in vacancies). This figure indicates that, excluding sources with very small quarterly counts of postings (less than 100), there is no relationship between counts of postings and prediction errors, supporting our hypothesis.

Given this finding, it makes sense to design a measure where each source exceeding some minimum number of postings (in this case 100) is given equal weight in estimating changes in vacancies. This measure would make more efficient use of the signals in the data (equal weight for signals of equal accuracy) and be robust to aberrations in the sources with the largest counts of postings (which might not reflect underlying changes in vacancies, as discussed above). The next section describes our proposed implementation of this type of measure.

### Algorithm

We use the following algorithm to estimate the percentage change in job vacancies between time points *t* − 1 and *t*. In this algorithm, time point *t* is the date of the Job Vacancies Survey and (in the job postings data set) the six-week period preceding the survey date. The algorithm uses changes in job postings between time periods *t* − 1 and *t* to estimate the change in job vacancies according to the official survey data captured at these two time points. This approach enables the relation of a flow variable (job postings) to a stock variable (job vacancies).

1. Identify all sources *j* = 1,…,*J* that had at least 100 job postings in each period (*t* − 1 and *t*).
2. Compute the percentage change in job postings for each source in this set:
  $$\Delta x_{jt} = \frac{{x_{jt} - x_{j,t - 1}}}{{x_{j,t - 1}}}$$
  where *x*_*jt*_ is the *j* th source’s count of postings in period *t*.
3. Apply *k*% winsorization (dampening of extreme values) to the set of Δ*x*_*jt*_ to obtain winsorized values $\Delta x_{jt}^{\left( w \right)}$.
4. Return the mean of these winsorized values $\Delta \bar x_{jt}^{\left( w \right)}$ as the estimate of the percentage change in job vacancies between time points *t* − 1 and *t*.

Applying *k*% winsorization in step 3 of the algorithm involves the following process:

1. Order the Δ*x*_*jt*_ from smallest to largest.
2. Identify the Δ*x*_*jt*_ corresponding to the *k*th and (1 − *k*)th percentiles of the ordered values: $\Delta x_{jt}^{\left( k \right)}$ and $\Delta x_{jt}^{\left(1 - k \right)}$ . Set these values as cut-offs.
3. Treat the extreme values by setting them at the relevant cut-off:
  - If $$\Delta x_{jt} \,<\, \Delta x_{jt}^{\left( k \right)}$$, set $$\Delta x_{jt}^{\left( w \right)} = \Delta x_{jt}^{\left( k \right)}$$.
  - If $$\Delta x_{jt} \,>\, \Delta x_{jt}^{\left( {1 - k} \right)}$$, set $$\Delta x_{jt}^{\left( w \right)} = \Delta x_{jt}^{\left( {1 - k} \right)}$$.

A key feature of our algorithm is that it treats each source’s percentage change in job postings as an equally weighted signal of the change in job vacancies (subject to winsorization). This feature is in response to our finding in the previous section that the sources with relatively small counts of postings are as accurate as sources with much larger counts of postings in predicting the change in vacancies.

Another key feature of our algorithm is the treatment of extreme values via winsorization. Sometimes sources have extremely large prediction errors (e.g., over 100 percentage points), as shown in Fig. 2. These extreme values can have an outsized effect on the mean percentage change across all sources, causing our measure to produce inaccurate estimates. We therefore use winsorization to reduce the influence of these extreme values on estimates. Winsorization reduces the influence of extreme values without removing them, unlike alternative methods such as trimming, and so delivers robust estimates whilst retaining some information from the extreme values (Ghosh & Vogt, 2012; Tukey, 1962). Winsorization is commonly used to treat outlier responses in surveys (Lee et al., 2011) and is implemented in several software packages for statistical analysis (Mair & Wilcox, 2020; Signorell et al., 2019).

**Fig. 3:**
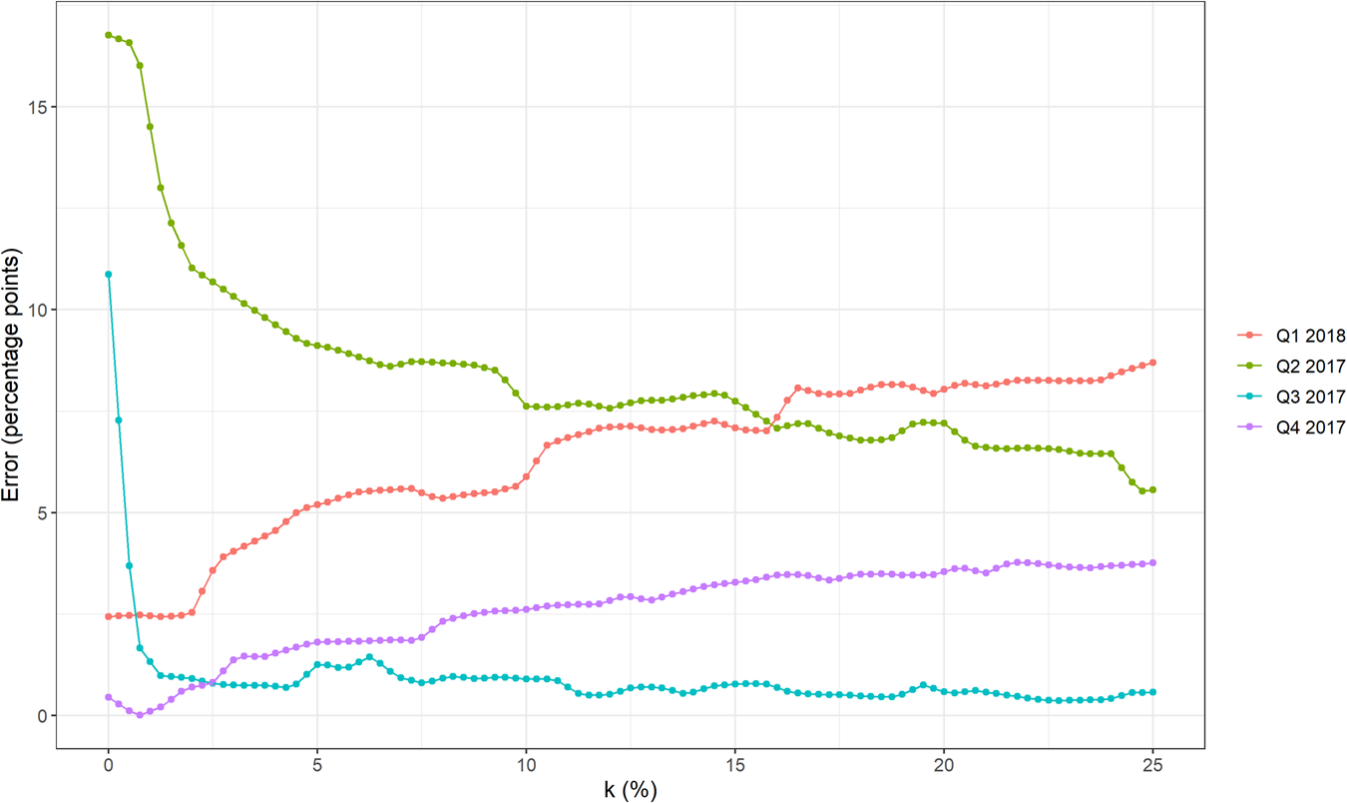
Winsorization and predictive accuracy. The algorithm’s absolute prediction errors under different levels of winsorization.

To choose the level of winsorization for our algorithm, we compute the algorithm’s absolute prediction errors under different levels of winsorization across four quarters of data (May 2017 to February 2018). We then choose the minimum level of winsorization that avoids the largest prediction errors. Figure 3 shows the prediction errors for each quarter under the different levels of winsorization. The figure shows that the second and third quarters of 2017 have large prediction errors without winsorization, and that these prediction errors are significantly reduced at winsorization levels *k* of 5–15%. We therefore test our algorithm under three levels of winsorization: 5%, 10% and 15%.

In our algorithm, the choices of the level of winsorization and the minimum number of job postings a source must have to be included in the ensemble are dependent on the data. As such, the choices of these parameter values will vary when the algorithm is applied to different data sets. The methods we have used above can be applied to guide the choices of these parameter values for different data.

### Study design

To evaluate our algorithm’s performance, we compare its estimates (which are made 6 weeks prior to the publication of Job Vacancies Survey estimates) to the estimates in the Job Vacancies Survey. We also compare our algorithm’s performance to the standard approach of using changes in the total count of job postings (across all sources) to estimate changes in vacancies.

We have data on changes in job postings from May 2017 to August 2022. We use data from May 2017 to February 2018 as training data to design our algorithm, as discussed in the previous section. We then use data from May 2018 to August 2022 as the test set to evaluate the algorithm’s performance. Here, we apply the algorithm to estimate changes in vacancies across the 18 quarters from May 2018 to August 2022, and then evaluate the accuracy of these estimates.

## Results

The algorithm provides accurate predictions of the changes in vacancies across the 18 quarters in our test set. Table 1 shows that the algorithm’s predicted percentage changes in vacancies in the different regions are highly correlated with the percentage changes in vacancies in the Job Vacancies Survey across the 18 quarters. For example, at the national level the algorithm’s predicted changes in vacancies and the actual changes in vacancies have a correlation coefficient of 0.95–0.96 (compared to 0.65 for the method that uses raw counts of postings to estimate changes in vacancies). The table also shows that the algorithm achieves similar results when applied separately to data on each of Australia’s largest states, though its performance tends to deteriorate as the size of the size of the ensemble (the number of sources to average over) decreases. It is not feasible to apply the method to Australia’s smallest states and territories (Tasmania, Northern Territory, and Australian Capital Territory) as these regions frequently have very small ensemble sizes (only one in some quarters), preventing any averaging across multiple signals.

**Table 1:** The methods’ correlations with the Job Vacancies Survey estimates across the 18 quarters from May 2018 to August 2022.

Level of estimates	Quarterly ensemble size (and range)	Raw job postings	Sites ensemble (*k* = 5%)	Sites ensemble (*k* = 10%)	Sites ensemble (*k* = 15%)
Australia	132 (68–167)	0.65	0.95	0.95	0.96
New South Wales	49 (21–70)	0.69	0.85	0.85	0.86
Victoria	44 (12–62)	0.77	0.92	0.92	0.92
Queensland	30 (13–43)	0.54	0.84	0.86	0.89
Western Australia	16 (6–23)	0.71	0.84	0.86	0.88
South Australia	10 (4–15)	0.52	0.74	0.75	0.77

Table 2 shows that the algorithm delivers significantly smaller mean absolute prediction errors than the raw counts approach across the 18 quarters. Here, we define the absolute error in each quarter as the difference between the algorithm’s estimated percentage change in vacancies and the actual change in job vacancies reported in the Job Vacancies Survey.

**Table 2:** The methods’ mean absolute prediction errors across the 18 quarters from May 2018 to August 2022.

Level of estimates	Raw job postings	Sites ensemble (*k* = 5%)	Sites ensemble (*k* = 10%)	Sites ensemble (*k* = 15%)
Australia	14.1	4.7	4.8	5.6
New South Wales	15.3	11.2	11.0	10.6
Victoria	13.1	6.8	6.8	7.0
Queensland	14.3	9.2	8.9	8.1
Western Australia	14.9	11.6	11.1	10.1
South Australia	20.5	17.8	17.0	16.4

**Fig. 4:**
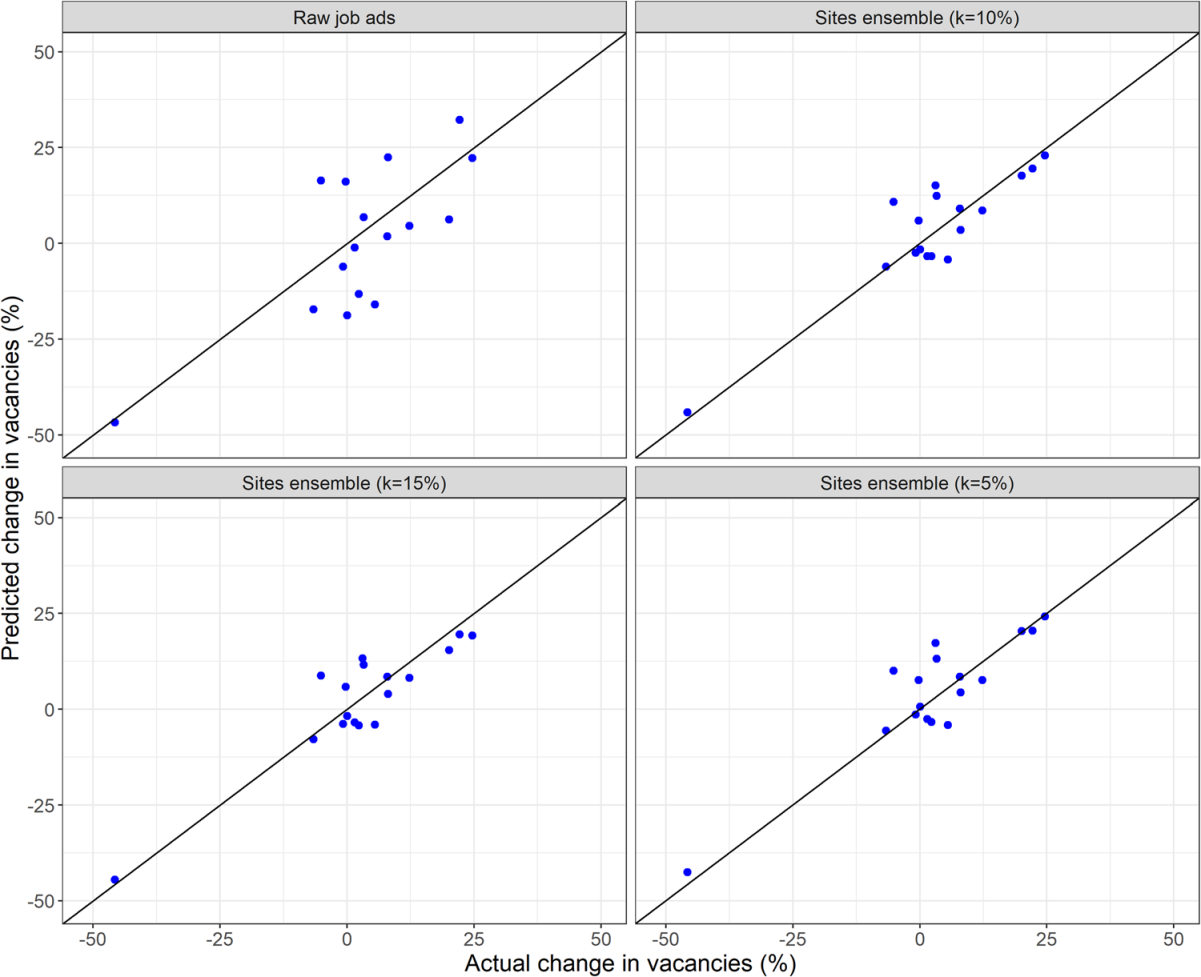
Actual and predicted changes in vacancies. The change in job vacancies according to the Job Vacancy Survey and each method’s predicted change in vacancies across the 18 quarters in the test set at the Australia level.

Figure 4 compares the changes in vacancies according to the Job Vacancies Survey to each algorithm’s estimated changes in vacancies across the 18 quarters in the test set at the Australia level. The figure shows that our algorithm accurately captures many of the large quarterly changes in vacancies that occurred over the test period. The figure also shows that the raw counts approach misses many of these quarterly changes in vacancies by a large margin.

**Fig. 5:**
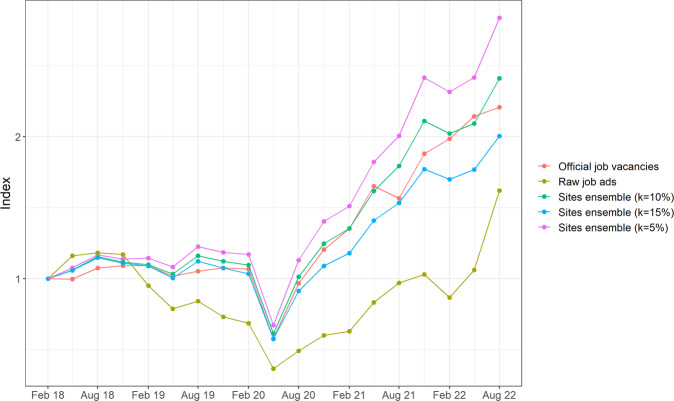
Actual and predicted index of job vacancies over time. Each method’s vacancies index compared to the Job Vacancies Survey’s official vacancies index at the Australia level.

Figure 5 compares each algorithm’s index of job vacancies to the Job Vacancies Survey’s index of job vacancies from the February 2018 quarter to the August 2022 quarter at the Australia level. We have set the indexes at 1 in February 2018 to visualise the differences between them over time. The figure shows that our algorithm provides a reasonably accurate approximation of the trajectory of vacancies across this 4.5-year period, particularly compared to the raw job postings approach.

**Fig. 6:**
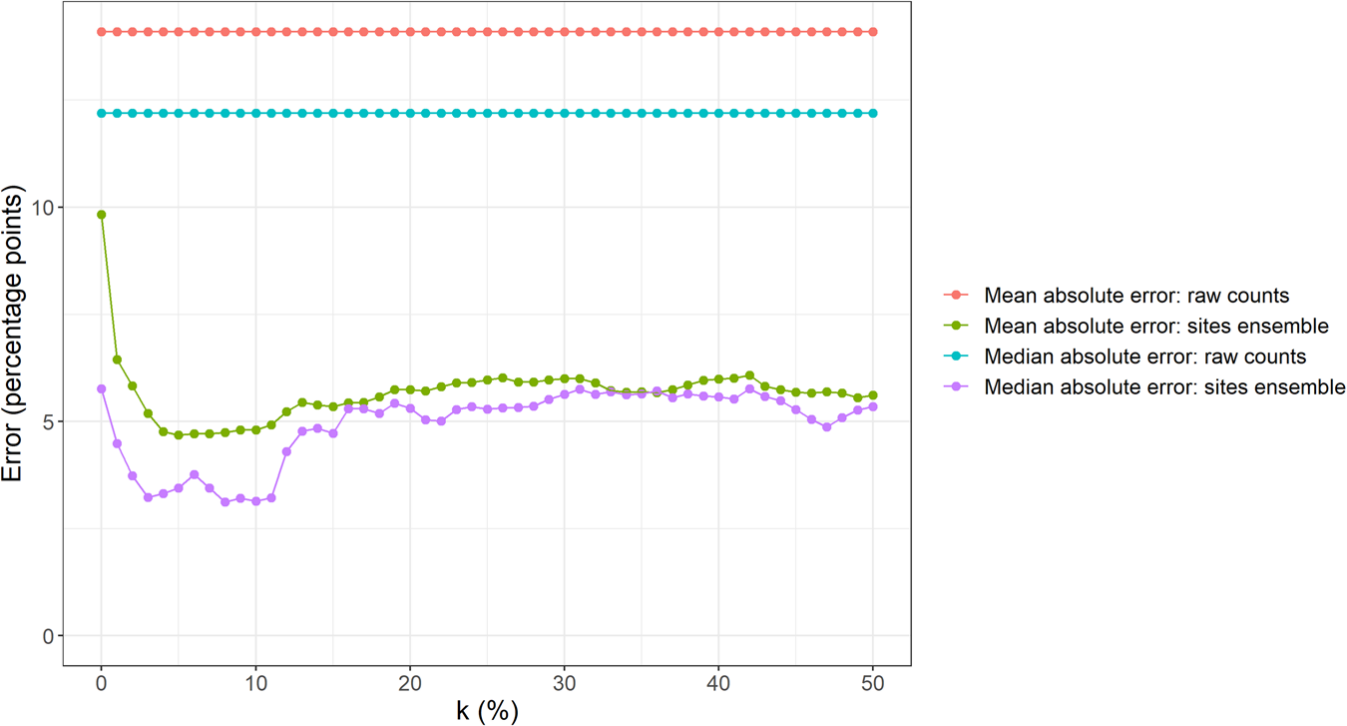
The algorithm’s predictive accuracy under different levels of winsorization. The algorithm’s average absolute errors at the Australia level under different levels of winsorization compared to the average absolute errors of estimates based on the raw counts of postings.

Our results are reasonably robust to the algorithm’s level of winsorization. Figure 6 shows the algorithm’s average absolute errors at the Australia level across the 18 quarters in the test set under different levels of winsorization: $k \in \{ 0\% ,\,1\% ,\, \ldots ,\,50\% \}$. Note that *k* = 50% is equivalent to using the median percentage change in postings across all sources to measure the actual percentage change in vacancies. The figure shows that the algorithm’s average prediction errors are smaller than those under the raw counts method regardless of the level of winsorization. This finding indicates that assigning an equal weight to each source’s signal is an improvement on the raw counts approach, even when the extreme values are left untreated (*k* = 0%). Figure 6 also shows that a winsorization level *k* of about 5–10% delivers the most accurate estimates across the test period.

## Discussion

### Robust signal averaging to estimate changes in job vacancies

Our study finds that unit weighting counts of job postings from different sources (treating each source as an equally reliable estimate of the population mean) significantly improves the accuracy with which online job postings can be used to predict official job vacancy statistics. We have shown that this method is significantly more accurate than the commonly applied method of using raw counts of job postings to predict changes in vacancies. The benefit of our method over traditional survey-based measures of vacancies is twofold: the estimates are delivered earlier (6 weeks before the publication of official vacancies estimates) and at a significantly higher frequency (as they can be updated daily, weekly etc.). Therefore, the method can provide earlier detection of changes in labour market conditions to inform policymakers’ responses.

The main idea behind our method is to assign equal weights to the signals of the change in vacancies from all the sources that Adzuna Australia collates (subject to winsorization). To apply our method, providers of online job postings data such as Burning Glass and Indeed (Kennedy, 2021) would need to capture and share data on the sources of their job postings. Providers likely already capture these data but do not share them as part of their standard products. Given the growing number of online job postings aggregators and the increasing use of job postings data to monitor labour market conditions in different countries and regions, our algorithm likely has widespread relevance for improving the accuracy of this monitoring. More generally, our algorithm provides a simple approach to transforming big time series data drawn from multiple sources of varying reliability into an accurate signal of the underlying quantity of interest. As such, the algorithm could be useful in a range of applications, such as using data on jobseekers’ online job search activity to measure labour supply (Faberman & Kudlyak, 2016).

### Limitations

Our algorithm is somewhat reliant on recruitment agencies’ posting behaviour and platforms’ scraping procedures remaining reasonably constant over time. Increased (decreased) activity from recruitment agencies could lead to increased (decreased) duplication of job postings that does not reflect the underlying change in vacancies (Office for National Statistics, 2021). Changes in platforms’ scraping and deduplication procedures can have a similar effect. Since our algorithm assigns equal weights to all the sources that Adzuna scrapes, its estimates are robust to changes in recruiters’ behaviour and platforms’ scraping procedures that only affect a few of the sources. The algorithm’s accuracy then decreases as the number of affected sources increases.

### Further research

There are several avenues for further research in this area. One avenue would involve comparing our algorithm to an algorithm where the weight assigned to each source is proportional to the source’s predictive accuracy in recent time periods. This type of weighting scheme would make the algorithm more closely fit (and perhaps overfit) recent data; whether this approach delivers greater accuracy is an empirical question. Further research is also needed to develop a method that provides reliable estimates of vacancies at finer levels of aggregation, such as in smaller regions or industries. We were unable to apply our method to some of the smaller regions in Australia due to there being an insufficient number of sources to average over. A different type of ensemble or method is required to measure vacancies at these finer levels of aggregation.

## Conclusion

In this paper we demonstrate a method for calibrating online job postings so that they can provide accurate and timely insight into changing labour market conditions. To support this type of analysis, job postings providers need to provide data on the source of each job posting. With such data and the method we present here, policymakers will be able to monitor labour market conditions in near real-time and adapt policy more rapidly in response to changing economic circumstances.

## Supplementary information

Supplementary Information

Supplementary Information

Supplementary Information

## Data Availability

The job postings data used in this study were provided by Adzuna Australia and are not publicly available. The job vacancies data used in this study are publicly available at https://www.abs.gov.au/statistics/labour/jobs/job-vacancies-australia/latest-release.
