# Supplementary material for: An algorithm for predicting job vacancies using online job postings in Australia
Source: Humanit Soc Sci Commun. 2023 Mar 13;10(1):102. doi: 10.1057/s41599-023-01562-9 (PMC10009847; doi:10.1057/s41599-023-01562-9)
Supplement: Supplementary file 1 — Supplementary Information [file 41599_2023_1562_MOESM1_ESM.docx]

**Supplementary Information**

**S1: Simulating and measuring stocks and flows of job postings**

In this study we have used the flow of new postings to the Adzuna database in the six weeks before the Job Vacancies Survey date as the indicator of the stock of vacancies on that date. Here, we use simulation to verify that increases in the time required to fill vacancies leads to increases in the flow of ‘new’ postings to the Adzuna database. We also show that this flow of postings is positively correlated with the stock of vacancies when the time required to fill vacancies changes. These findings confirm that the flow of new postings to the Adzuna database is a reasonable measure of the stock of vacancies.

We simulate the following job postings and measurement process across days $t=1, \ldots, T$:

1. The number of new postings on day $t$ is drawn from the $Poi(1000)$ distribution, which gives a mean of 1,000 postings per day. Index the postings by $i=1, 2, \ldots$
2. Each posting’s duration $d_{i}$ (time to fill) is drawn from from a $Weibull(\theta, 3)$ distribution, where the scale parameter $\theta$ largely determines the mean duration.
3. Assign each new posting a date on which it is filled and removed: $f_{i}=t+d_{i}$.
4. The true stock of live postings then contains all postings with $f_{i}>t$.
5. The platform scrapes any ‘new’ postings from the stock each day, where a ‘new’ posting is any posting that is not already in the platform’s database.
6. The platform then cleans its database by removing any posting:
   1. With an end date of over 30 days ago; or
   2. That has been in the database for over 60 days.

We then measure the following quantities on day $T$:

- The true stock of postings, which contains any posting with a fill date beyond the current date.
- The platform’s stock of postings, which contains all postings in the platform’s database.
- The true flow of new postings in the 6 weeks preceding day $T$, which is the number drawn in step 1 summed across the previous 6 weeks.
- The flow of new postings to the platform in the 6 weeks preceding day $T$, which is the number of new postings from step 1 plus the postings that the platform removes in step 6b and then scrapes back in the next day in step 5 summed across the previous 6 weeks. These latter postings are postings that the platform removed after 60 days but had not been filled and were still ‘live’ in the true stock.

We simulate a range of different values for $\theta$ to show the effect of changes in job posting duration (or time required to fill the posting) on the above flows and stocks of postings. Figure S1 shows the results of this simulation. The horizontal axis shows the mean days to fill the job vacancy, which is closely related to $\theta$. The figure shows that increases in the mean days required to fill vacancies lead to increases in both the true stock of postings on day $T$ and the flow of new postings to the platform in the 6 weeks prior to day $T$.

**S2: Raw counts of job postings and job vacancies**

Figure S2 provides the counts of job postings and vacancies we used to create the indexes in Figure 1. The number of vacancies is the Job Vacancies Survey estimate of vacancies on each survey date. The number of job postings is the total number of new postings in the six weeks preceding the survey date.
