# Supplementary figures and images for: An algorithm for predicting job vacancies using online job postings in Australia
Source: Humanit Soc Sci Commun. 2023 Mar 13;10(1):102. doi: 10.1057/s41599-023-01562-9 (PMC10009847; doi:10.1057/s41599-023-01562-9)

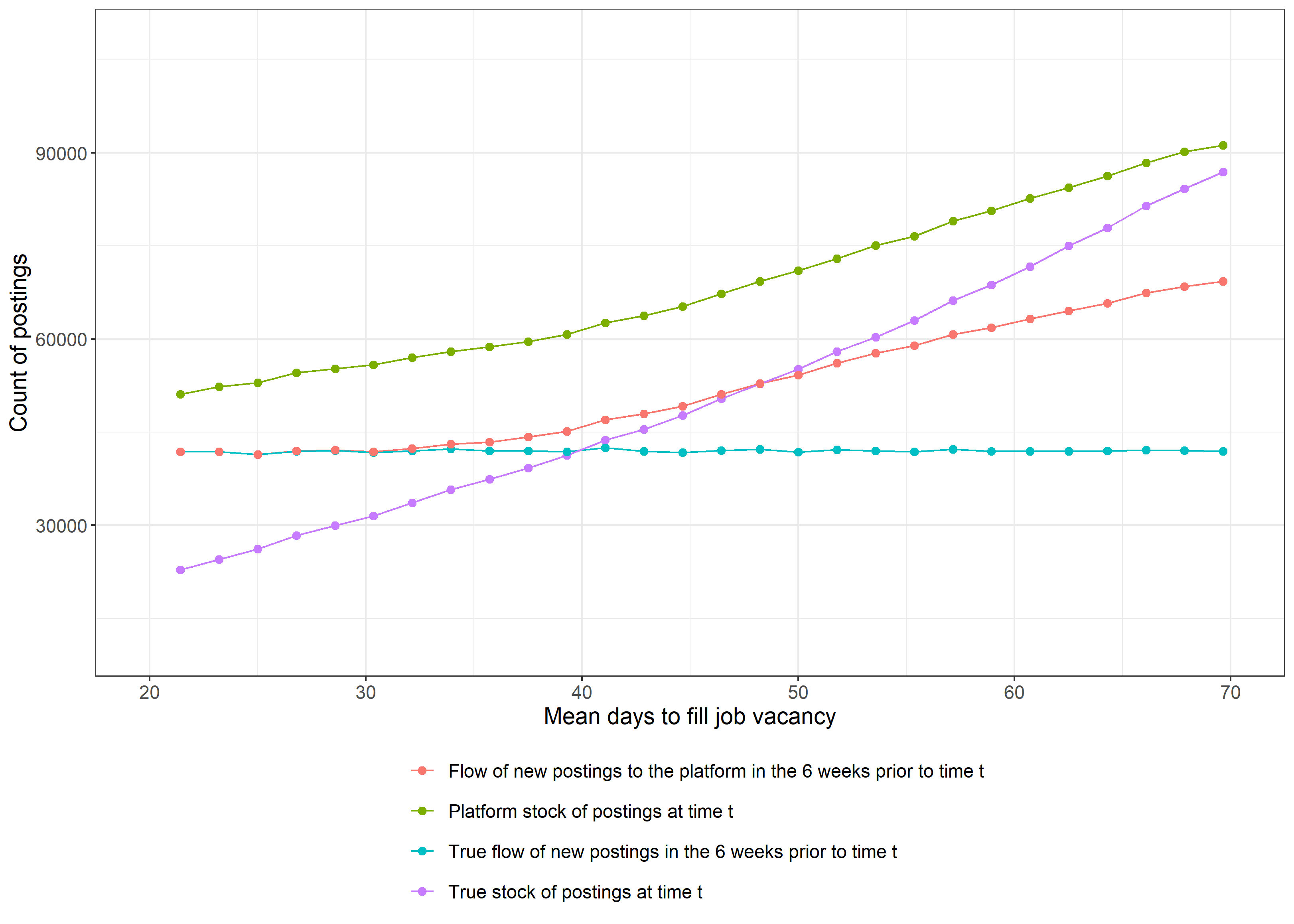

Supplement: Supplementary file 2 — Supplementary Information [file 41599_2023_1562_MOESM2_ESM.png]

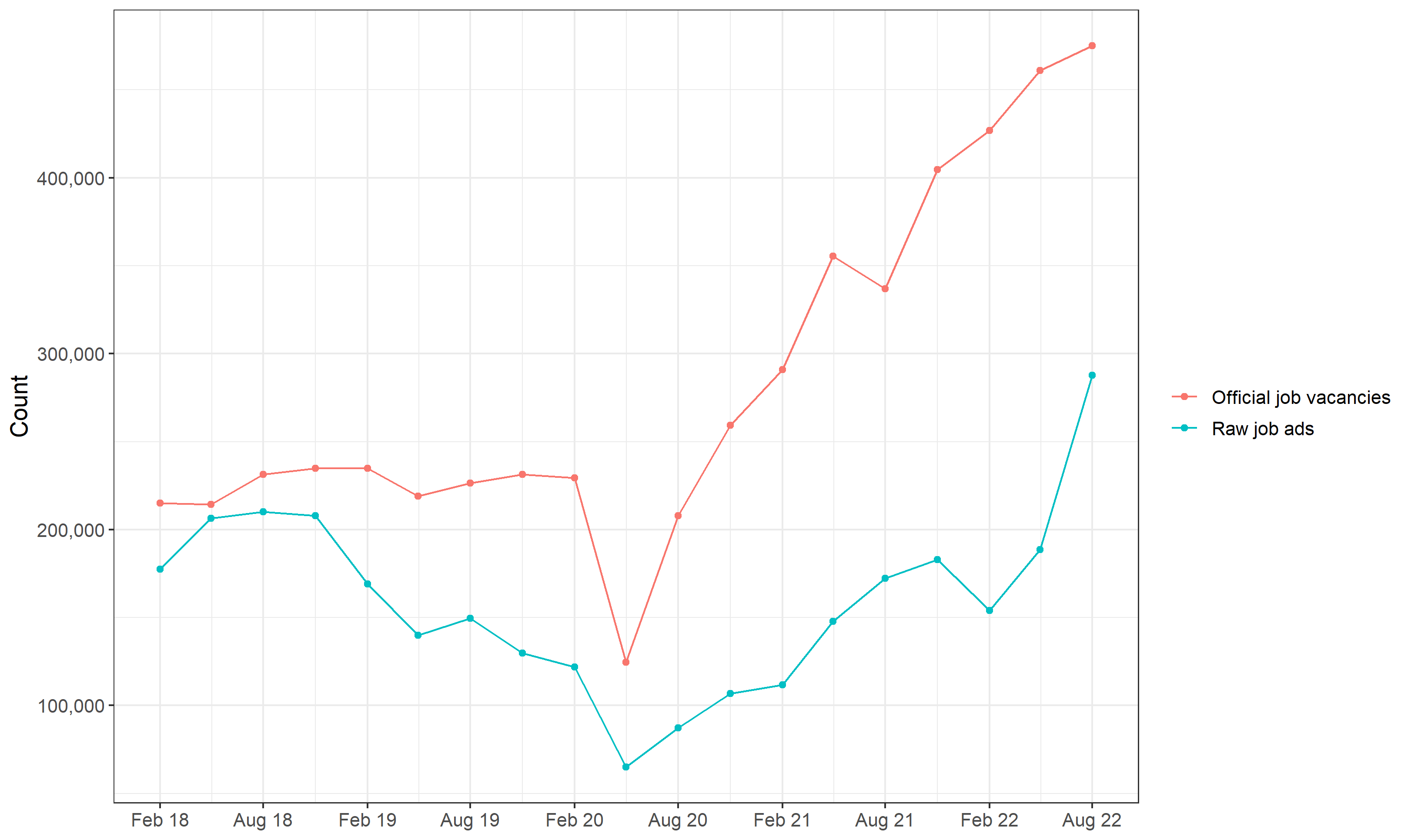

Supplement: Supplementary file 3 — Supplementary Information [file 41599_2023_1562_MOESM3_ESM.png]
